# Effect of serrated grain boundary on tensile and creep properties of a precipitation strengthened high entropy alloy

**DOI:** 10.1080/14686996.2022.2158043

**Published:** 2023-01-10

**Authors:** Jhuo-Lun Lee, Pei-Te Wang, Kai-Chi Lo, Pai-Keng Shen, Nien-Ti Tsou, Koji Kakehi, Hideyuki Murakami, Che-Wei Tsai, Stéphane Gorsse, An-Chou Yeh

**Affiliations:** a High Entropy Materials Center, National Tsing Hua University, Hsinchu, Taiwan (ROC); b Program in Prospective Functional Materials Industry, National Tsing Hua University, Hsinchu, Taiwan (ROC); c Department of Materials Science and Engineering, National Tsing Hua University, Hsinchu, Taiwan (ROC); d Department of Materials Science and Engineering, National Yang Ming Chiao Tung University, Hsinchu, Taiwan (ROC); e Department of Mechanical Engineering, Tokyo Metropolitan University, Hachiojishi, Tokyo, Japan; f Research Center for Structural Materials, National Institute for Materials Science, Tsukuba, Japan; g Department of Nanoscience and Nanoengineering, Waseda University, Shinjuku, Tokyo, Japan; h CNRS, University of Bordeaux, Bordeaux INP, ICMCB, UMR 5026, Pessac, France

**Keywords:** Selective laser melting, high-entropy alloys, hot ductility drops, tensile creep properties, serrated grain boundary

## Abstract

In this study, tensile and creep deformation of a high-entropy alloy processed by selective laser melting (SLM) has been investigated; hot ductility drop was identified at first, and the loss of ductility at elevated temperature was associated with intergranular fracture. By modifying the grain boundary morphology from straight to serration, the hot ductility drop issue has been resolved successfully. The serrated grain boundary could be achieved by reducing the cooling rate of solution heat treatment, which allowed the coarsening of L1_2_ structured γ′ precipitates to interfere with mobile grain boundaries, resulting in undulation of the grain boundary morphology. Tensile and creep tests at 650°C were conducted, and serrated grain boundary could render a significant increase in tensile fracture strain and creep rupture life by a factor of 3.5 and 400, respectively. Detailed microstructure analysis has indicated that serrated grain boundary could distribute strains more evenly than that of straight morphology. The underlying mechanism of deformation with grain boundary serration was further demonstrated by molecular dynamic simulation, which has indicated that serrated grain boundaries could reduce local strain concentration and provide resistance against intergranular cracking. This is the first study to tackle the hot ductility drop issue in a high-entropy alloy fabricated by SLM; it can provide a guideline to develop future high-entropy alloys and design post heat treatment for elevated temperature applications.

## GRAPHICAL ABSTRACT

**Figure:**
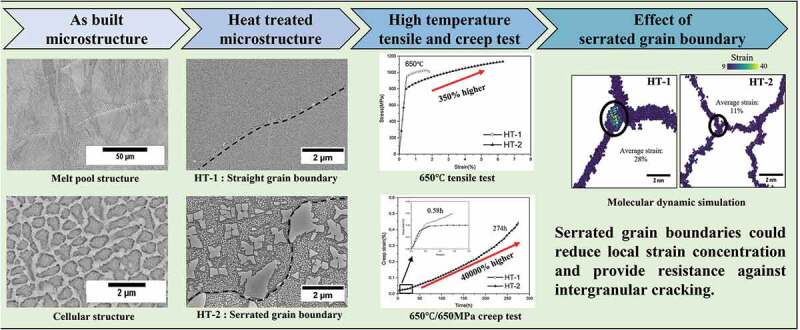

## 1. Introduction

High-entropy alloys (HEAs) are potential structural materials that have shown promising structural properties [1–7] and have been subjected to various manufacturing processes [8–13], including selective laser melting (SLM) [14–17]. HEAs fabricated by SLM are shown to possess excellent mechanical strength at room temperature [14,17–22], and there is a limited number of reports on their high temperature mechanical properties [14,23]. In fact, on the subject of high temperature mechanical properties of HEAs in general, several studies have reported a phenomena of hot ductility drop [23–33]. Otto et al. [24] investigated CoCrFeMnNi and observed a tensile strain drop by 40% at 400°C-600°C compared to that at room temperature. A 50% loss of strain at 400°C was reported by Wu et al. [25] for a series of alloys based on CoCrFeMnNi. Lin et al. [23] studied NiFeCoCr and showed a decrease of tensile fracture strain to about 5% at 600°C-700°C. Chang et al. [28] investigated Al_3.3_1Co_27_Cr_18_Fe_18_Ni_27.27_Ti_5_ and reported a drop of tensile strain by 75% at 750°C compared to that of room temperature. It has been proposed that nano-clustering of voids would generate micro-holes at grain boundaries of HEAs during high-temperature deformation and caused ductility loss [34]. Hot ductility loss was also a phenomenon observed in several traditional alloys [35–37] and thermos-mechanical processes have been applied to modify their grain boundary characteristics in order to improve ductility at elevated temperatures [38–41]. However, thermo-mechanical process is not applicable as a post processing step for SLM, where components are usually built closely to the final dimension. Another possible approach to resolve the hot ductility loss is to introduce serrated grain boundary (SEGB) [42–47], it usually involves a post heat treatment step to induce interactions between mobile grain boundaries and second phases, including carbides [45], δ phase [46], or γ′ phase [47]. SEGB has been reported to impede intergranular crack propagation [46,48,49], improve the tensile properties of Ni-based superalloys fabricated by SLM [50], and also the ductility of Ni_46.23_Co_23_Cr_10_Fe_5_Al_8.5_Ti_4_W_2_Mo_1_C_0.15_B_0.1_Zr_0.02_ fabricated by arc melting [51]. In this study, we have unprecedentedly utilized SEGB on an HEA fabricated by SLM. This work is the first to investigate the underlying mechanism of hot ductility drop of a HEA fabricated by SLM and study the effect of SEGB on its high temperature tensile and creep properties.

## 2. Material and methods

### 2.1 Alloy of interest and powder preparation

**Figure 1.**
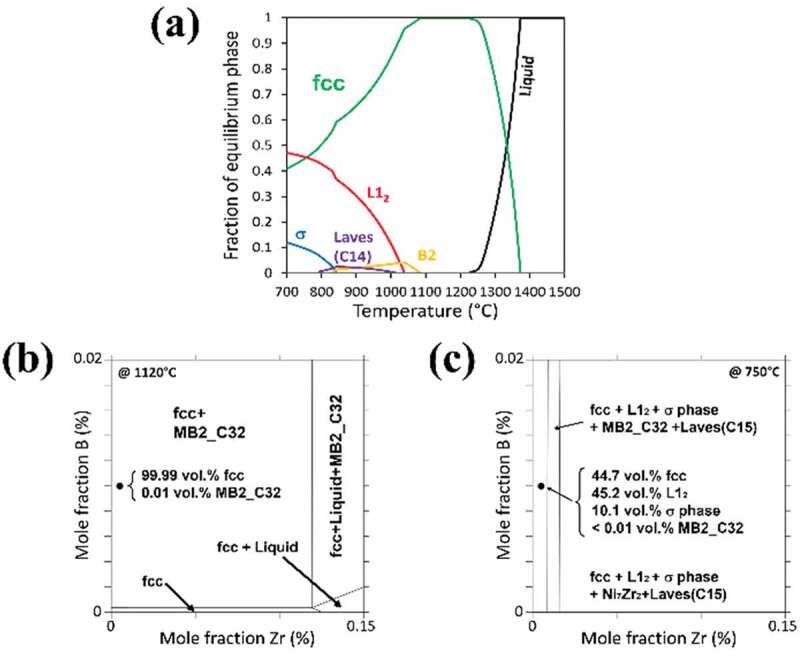
Simulated phase diagrams, (a) Equilibrium phase diagram. Note that the amount of boride is too small to be shown. (b) Isothermal prediction at 1120°C, illustrating the solubility of Zr and B. (c) Isothermal prediction at 750°C.

In this study, Al_8_Co_35_Cr_18_Ni_34_Ti_3_Nb_2_Zr_0.005_B_0.01_ (at.%) (designated as SLMHEA) was designed based on a previously reported HEA – Al_3.3_1Co_27_Cr_18_Fe_18_Ni_27.27_Ti_5_ (at.%), which was found to exhibit hot ductility drop phenomena [28]. Thermo-Calc software [52] based on the CALPHAD method with the TCHEA4 database was used to simulate the equilibrium and isothermal phase diagrams. Figure 1(a) shows that SLMHEA possesses mainly fcc-structured γ matrix and fcc-ordered L1_2_ structured γ′ precipitates. In the L1_2_ phase, Ni and Co atoms occupy the face centers and Al, Ti atoms are located at the corners of the unit cell [53]. Furthermore, sigma phase (σ), C_14_ laves phase, B2 phase and MB2_C32 phase have been predicted. The σ phase possesses tetragonal structure and has a high content of Cr and Mo [54], C_14_ laves phase has a hexagonal structure with a typical composition of (FeCoCrNi)_2_Nb [53], B2 is an ordered bcc phase typically enriched in Al, Ni and Co [55], and MB2_C32 is the TiB_2_ boride with hexagonal crystal structure [56]. According to Figure 1(b), the composition of SLMHEA is below the solubility limit of Zr and slightly above the solubility limit of B at 1120°C. Figure 1(c) shows that SLMHEA at 750°C is located in a 4-phase field including about the same fraction of fcc and L1_2_ structured phase as the main phases, plus about 10 vol.% of σ phase and less than 0.01 vol.% of TiB_2_.

Prealloyed powders were fabricated by gas atomization and supplied by Chung Yo Materials Co. Ltd., Kaohsiung, Taiwan (R.O.C.). The particle size distribution was analyzed by Mastersizer 3000 (Malvern Panalytical, UK), where d10, d50, and d90, were 27.4, 38.8, and 54.4 μm, respectively. Zr and B were added by 2D mixing the prealloyed powder with 0.01 wt.% of ZrB2 flakes (<5 μm in size).

### 2.2 SLM process

An in-house SLM machine equipped with the IPG YLR AC 500 W yttrium fiber laser system was used to fabricate specimens. The working chamber was purged and filled with pure argon to ensure that the oxygen content was below 100 ppm. Optimized SLM processing parameters were determined by experimental trials with an aim to achieve the density higher than 98% determined by the Archimedes method. The SLM processing parameters used in this work were as follows: laser power was 270 W, laser spot size was 58 μm, scanning speed was 1000 mm/s, and hatch distance was 75 μm. The scanning strategy was zigzag with a 67° rotation between each layer, and the thickness of each powder layer was 50 μm. A carbon steel base plate was used and was pre-heated to 200°C. Bulk samples with 14 × 85 × 8 mm^3^ were fabricated.

### 2.3 Heat treatments

**Figure 2.**
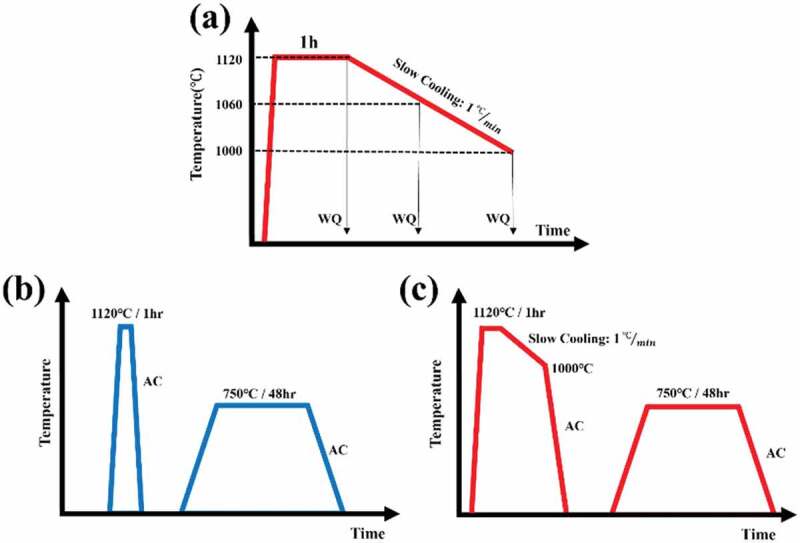
Schematic diagrams of heat treatments (a) interrupted cooling experiments, (b) HT-1, and (c) HT-2.

Based on the simulated phase diagram shown in Figure 1(a), the γ′ solvus temperature was 1080°C, and the B2 phase solvus temperature was 1100°C, so the solution heat treatment in this work was conducted at a temperature above the solvus of γ′ and B2, i.e. 1120°C for 1 h, and the aging heat treatment to induce γ′ precipitation was conducted at 750°C for 48 h. Furthermore, to study the interactions between γ′ particles and grain boundaries, interrupted cooling experiments with different terminating temperatures were conducted. The cooling rate after the solution heat treatment was set at 1°C/min. Samples were then water quenched at various temperatures, i.e. 1060°C, and 1000°C. The heat treatment history of the interrupted cooling experiments is shown in Figure 2(a). In this study, mechanical properties evaluations were based on samples subjected to two different heat treatment processes, i.e. HT-1, and HT-2. The corresponding heat treatment profiles of HT-1, and HT-2 are shown in Figure 2(b,c) respectively. The HT-1 process involved a solution heat treatment with air-cooling to room temperature followed by an aging heat treatment. The HT-2 heat treatment consisted of a solution heat treatment with slow cooling of 1°C/min to 1000°C, and then air-cooled to room temperature plus an aging heat treatment. All heat treatments were conducted by the Thermo Scientific™ (USA) Lindberg/Blue box furnace.

### 2.4 Microstructure characterization

Microstructures of SLMHEA were examined by scanning electron microscope (SEM, Gemini 300 Carl Zeiss (Germany) SEM and FE-SEM JEOL (Japan) JSM-7200), specimens were ground and polished with a 0.05 μm Al_2_O_3_ dispersion fluid, then electrolytically etched in 20 vol% phosphoric acid under 2 V for 2 s with a copper cathode. For electron back scattering diffraction (EBSD) analysis, an AZtec EBSD system (Oxford Instruments, UK) was utilized, samples were electropolished in 20 vol% perchloric acid +80 vol% ethanol under 20 V at −20°C for 20 s with a platinum cathode. Raw EBSD data were post-processed by the Aztec Crystal software [57]; kernel average misorientation (KAM) analysis, which has been applied to analyze the internal strain through quantifying local lattice curvature and distortion of crystalline materials in previous work [58], was used to evaluate internal strain distribution. The grain size was determined as the mean of the equivalent diameters of the grains, and the particle size was estimated by ImageJ software [59]. JEOL (Japan) JEM-F200 (200kV) high-resolution transmission electron microscope (TEM) was used to analyze phase constituents. The specimens for TEM analysis were ground by 2000 grit SiC paper to a thickness of 60 μm and then punched into round discs with a diameter of 3 mm. The discs were twin-jet polished in a 10 vol% perchloric acid +90 vol% ethanol solution under 20 V at −20°C.

### 2.5 Mechanical tests

Specimens for tensile tests were sectioned perpendicularly to the building direction and machined into the bone-shaped specimens with a gauge dimension of 19 × 3 × 1.5 mm^3^. All surfaces of specimens were ground with 2000 grit SiC sandpaper prior to tensile tests. Room temperature tensile tests were conducted at 25°C and a constant strain rate of 10^−3^ s^−1^ by the INSTRON 4468 tensile tester. High temperature tensile tests were performed at 650°C with a constant strain rate of 10^−3^ s^−1^ by the Shimadzu AGS-X 100kNX tensile tester. Specimens for creep tests were sectioned perpendicularly to the building direction and machined into the bone-shaped specimens with a gauge dimension of 16 × 3 × 3 mm^3^. All surfaces of specimens were ground with 2000 grit SiC sandpaper prior to creep tests. All creep tests were conducted at 650°C/650MPa by ATS Series 2330 Lever Arm Creep Testing System.

### 2.6 MD simulations

Molecular dynamic (MD) model was conducted to simulate the difference in strain distributions between triple junctions of straight grain boundaries and that of serrated grain boundaries. Atomsk [60], an open source command-line program manipulating atomic systems for the purposes of atomistic calculations in the areas of computational physics, was applied. An atomistic model of polycrystalline fcc nickel containing planar and serrated grain boundaries was created and loaded to directly observe the strain distribution. Three-dimensional surface profile of grain boundaries was converted from the experimental microstructure observations by using Dream.3D [61]. Periodic boundary conditions, commonly applied in molecular dynamics to avoid huge simulation sizes and improve simulation efficiency, were applied in all three directions of the model. The interatomic interactions were described using second nearest-neighbor modified-embedded-atom-method (2NN MEAM) potentials adjusted by Etesami et al [62]. The temperature of the model was firstly equilibrated at 650°C for 100 ps with the timestep of 2 fs. The loadings were then applied along the y-direction by stretching the model at a constant force of 650 MPa until fracture occurred. Both procedures were at an isothermal-isobaric NPT ensemble and calculated by LAMMPS [63]. The simulation results were visualized by OVITO [64], where the atomic volumetric strain and structures were determined by the Voronoi analysis modifier [64] and the common neighbor analysis (CNA) [65].

## 3. Results and analysis

### 3.1 As-prepared microstructure

**Figure 3.**
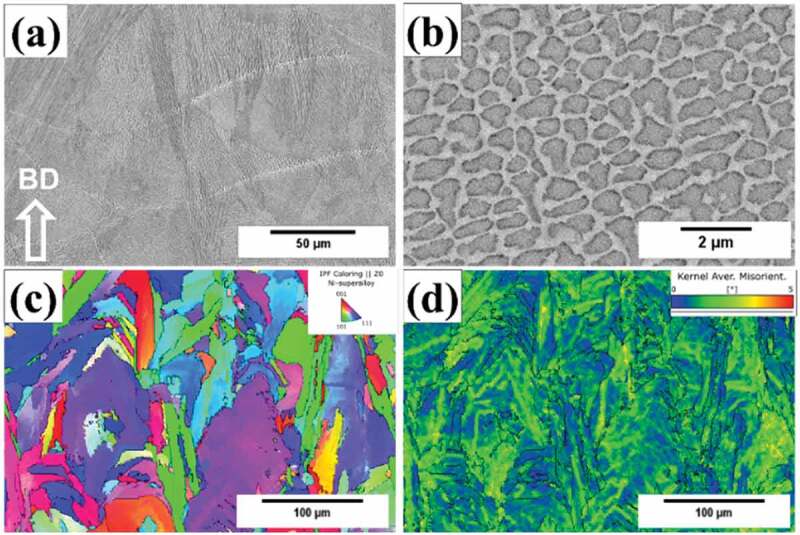
Microstructure of as-prepared sample. (a) Secondary electron image showing the melt pools. (b) Backscattered electron image showing the sub-micron cellular structures. (c) Inverse pole figure map showing the grain texture. (d) KAM map showing the residual strain.

The microstructures of the as-prepared samples are shown in Figure 3. At low magnification, melt pool structures are clearly visible (Figure 3(a)), and the microstructure contained sub-micron cellular dendritic structures with an average cell size of 692 nm (Figure 3(b)). The inverse pole figure map indicates that elongated columnar grains grew along the building direction (Figure 3(c)), and this could be related to the epitaxial grain growth between layers due to thermal gradient during the SLM process. The average grain size is estimated as 14.4 μm. Figure 3(d) shows that significant residual strains (green and yellow color regions) were accumulated inside the as-prepared sample, this could be associated with the thermal contraction during repeated fast cooling and heating process during SLM.

### 3.2 Microstructure evolution

**Figure 4.**
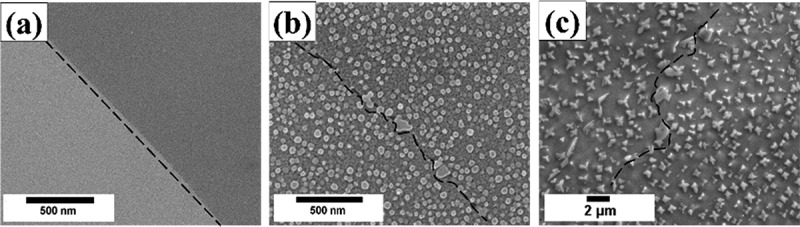
Interrupted cooling experiments. The dashed lines indicate the morphology of grain boundaries. Figures show the secondary electron images of (a) water quench after holding for 1h at 1120°C, (b) slow cooling to 1060°C, and (c) slow cooling to 1000°C.

To study the interactions between precipitates and grain boundary morphology, interrupted cooling experiment was conducted, and microstructure evolution was captured during slow cooling to various temperatures (Figure 4). After solution treatment for 1 h at 1120°C followed by water quenching, no precipitates could be observed (Figure 4(a)). With 1°C/min slow cooling to 1060°C, homogeneous distribution of spherical precipitates could be seen, and a slight distortion of grain boundary was observed (Figure 4(b)). With further cooling to 1000°C, blocky and floral precipitate could be identified, and serrations of grain boundary were clearly identified (Figure 4(c)).

**Figure 5.**
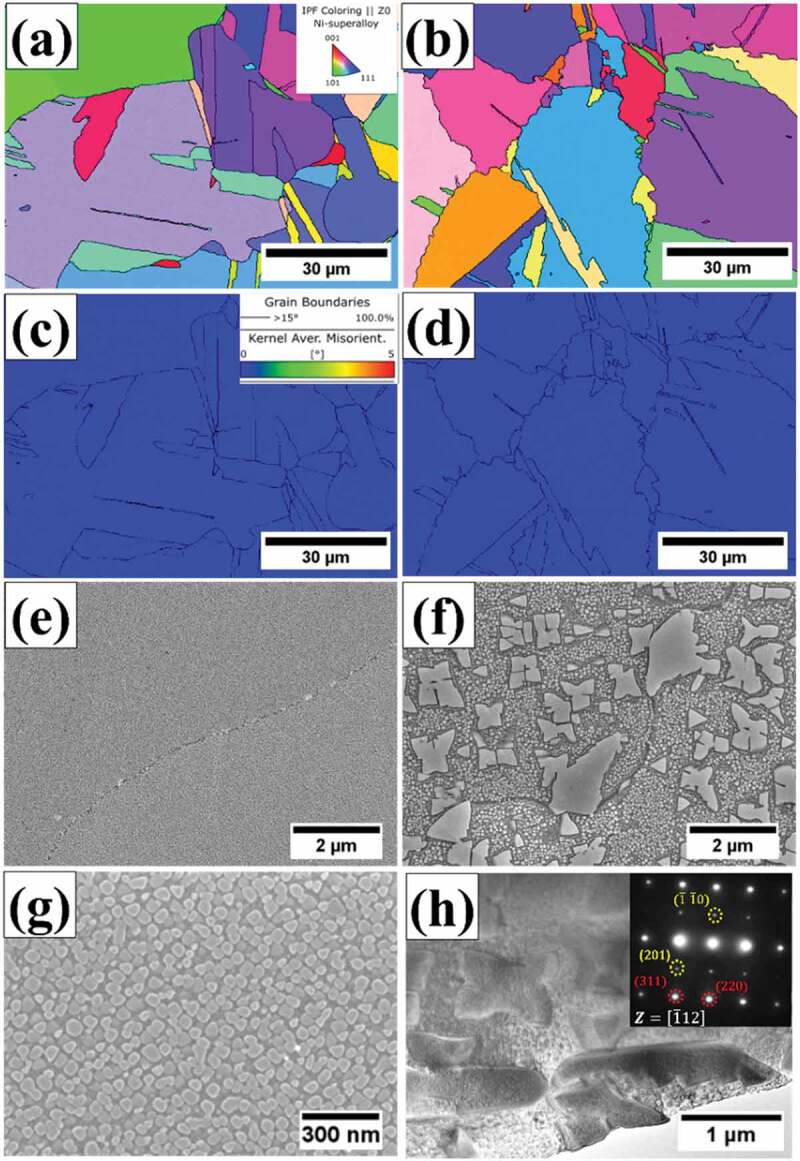
Microstructures of HT-1 sample (a, c, e, g) and HT-2 sample (b, d, f, h), where (a, b) are inverse pole figure maps, (c, d) are KAM maps, and (e, f, g) are SEM micrographs. The TEM image and diffraction pattern in (h) show the fcc matrix and L1_2_ precipitates.

Microstructure observations after HT-1 and HT-2 heat treatments are shown in Figure 5. For both HT-1 and HT-2 samples, microstructures contained a mixture of equiaxed grains (Figure 5(a,b)), and according to the KAM analysis, internal strains of the as-prepared sample were relieved (Figure 5(c,d)). The average grain sizes of HT-1 and HT-2 samples are 44.5 μm and 33.8 μm, respectively. The grain boundary of HT-1 sample was straight (Figure 5(e)), and the grain boundary morphology of HT-2 sample was serrated. The HT-2 sample contained coarsen precipitates with an average size of 1.8 μm (Figure 5(f)); by contrast, fine precipitate with an average size of 60.1 nm was dispersed in HT-1 sample (Figure 5(g)). For HT-2 sample, the average wavelength and amplitude of the grain boundary serration were 3.29 ± 0.38 μm and 1.69 ± 0.24 μm, respectively. Further analysis by TEM shows that the microstructure contained fcc plus L1_2_ structured superlattice diffraction spots, as shown in Figure 5(h). Therefore, it can be concluded that the phase constituents after heat treatments were fcc matrix plus L1_2_ γ′particles. In fact, there was no σ phase, C14 Laves phase, B2 phase, and borides observed experimentally. Comparing with the predicted phase diagrams shown in Figure 1, the discrepancy between simulations and experimental observations might be due to the inaccuracies in the current thermodynamic databases for HEAs, or the condition of samples in present work was far from the thermodynamic equilibrium.

### 3.3 Tensile properties

**Figure 6.**
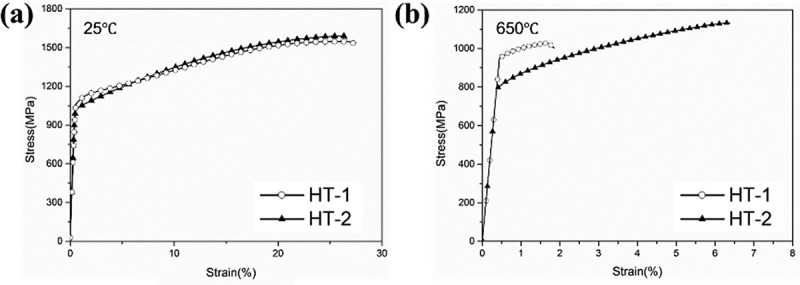
Tensile stress-strain curves at (a) 25°C and (b) 650°C.

**Figure 7.**
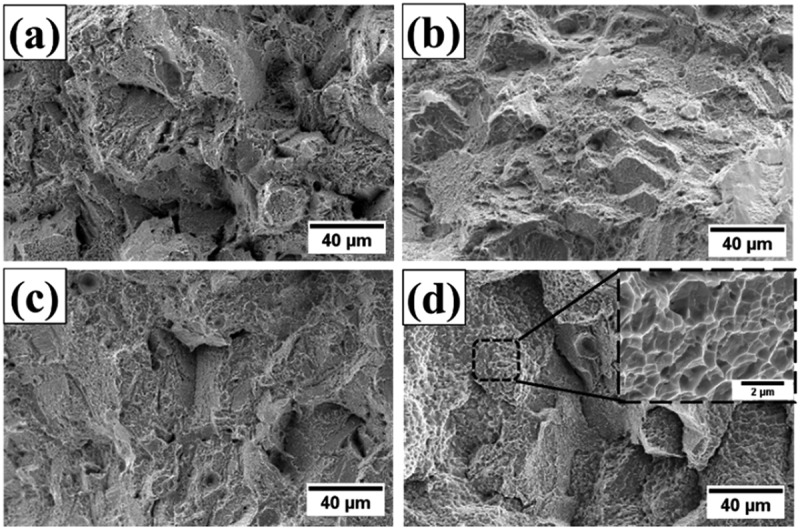
Tensile fracture surfaces of (a, b) HT-1 sample, (c, d) HT-2 sample. The testing temperatures were 25°C for (a, c) and 650°C for (b, d).

HT-1 and HT-2 samples were subjected to the tensile test at 25°C and 650°C. Figure 6 shows the engineering stress-strain curves, and the mechanical properties are summarized in Table 1. For the HT-1 samples, the yield strength (YS), ultimate tensile strength (UTS), and fracture strain (ε) were 1061 MPa, 1529 MPa, and 27.2% at 25°C, and 959 MPa, 1026 MPa, and 1.8% at 650°C, respectively; HT-1 samples showed a severe drop in tensile strain by 93%. For HT-2 samples, the YS, UTS, and ε were 996 MPa, 1589 MPa, and 26.4% at 25°C, and 803 MPa, 1131 MPa and 6.3% at 650°C, respectively. The room temperature tensile properties were similar for both heat-treated samples; at 650°C, the HT-2 sample exhibited an increase in tensile fracture strain by a factor of 3.5 with a minor expense of strength. Figure 7 shows the fracture surfaces of tensile tested samples. At 25°C, both HT-1 and HT-2 samples showed the typical ductile fracture with dimpling feature. At 650°C, HT-1 sample displayed flat and smooth fracture surfaces, which indicates limited plastic deformation (Figure 7(b)). By contrast, HT-2 sample presented rough surfaces and small dimples on the fracture surfaces (Figure 7(d)).

**Table 1.** Tensile properties of HT-1 and HT-2 samples.

	Temperature(°C)	YS(MPa)	UTS(MPa)	ε(%)
HT-1	25	1061	1529	27.2
	650	959	1026	1.8
HT-2	25	996	1589	26.4
	650	803	1131	6.3

### 3.4 Creep properties

**Figure 8.**
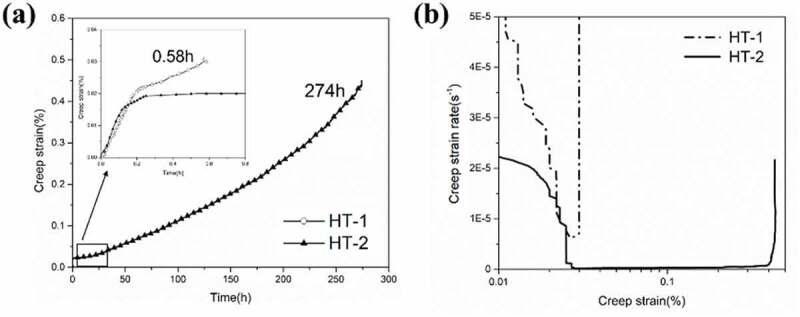
Creep rupture curves under 650°C/650MPa. (a) Plot of creep strain vs. time (b) creep strain rate vs. logarithmic creep strain.

Both HT-1 and HT-2 samples have been subjected to creep tests at 650°C/650MPa. As shown in Figure 8(a), the creep rupture life of HT-1 and HT-2 samples were 0.58 h and 274 h, respectively. HT-1 possessed a creep fracture strain of 0.03%, and HT-2 exhibited a 0.45% creep fracture strain. The plot of creep strain rate verse logarithmic creep strain is shown in Figure 8(b), and the minimum creep strain rates of HT-1 and HT-2 samples are determined to be 6.4 × 10^−6^ s^−1^ and 2.4 × 10^−7^ s^−1^, respectively. The creep curve of HT-1 sample showed a limited secondary creep region followed by a sharp onset of tertiary creep to failure. The creep curve of HT-2 sample showed a secondary creep region, followed by a pronounced tertiary creep region. It appears that SEGB in HT-2 sample had a significant influence on the high temperature creep deformation in terms of decreasing the minimum creep strain rate and prolonging the tertiary creep region.

**Figure 9.**
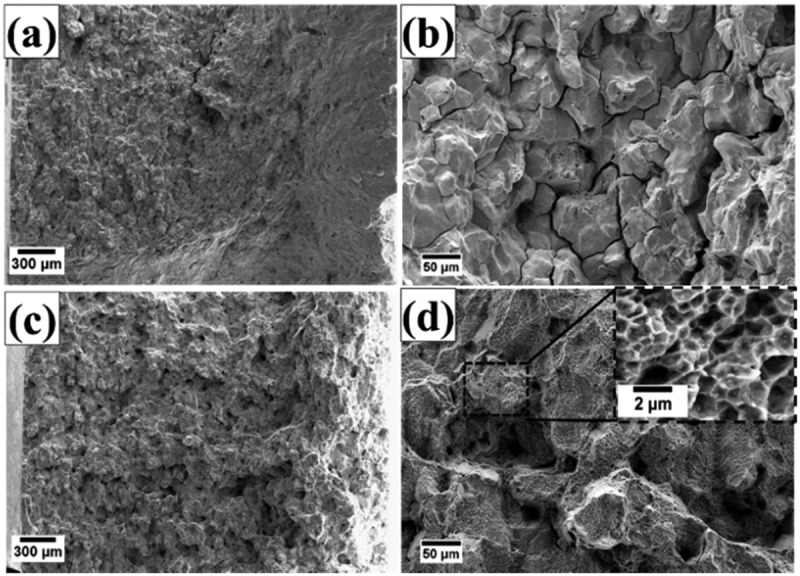
Fracture surfaces of crept samples. (a, b) HT-1 sample, and (c, d) HT-2 sample.

**Figure 10.**
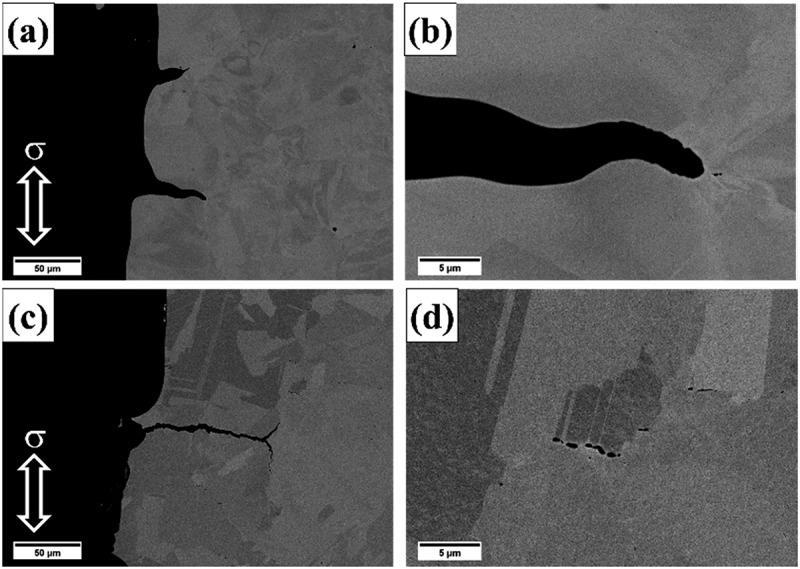
The longitudinal sections of crept samples. (a, b) HT-1, and (c, d) HT-2. The hollow white arrows represent the stress loading direction.

**Figure 11.**
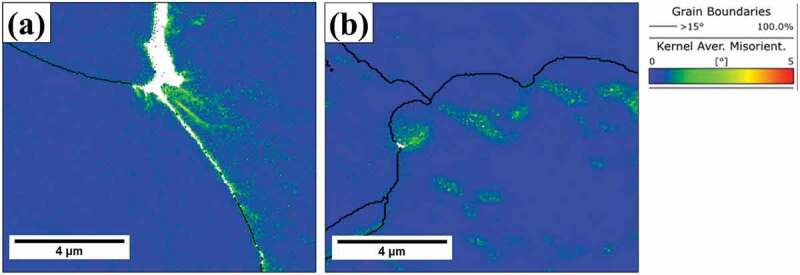
KAM analysis of crept samples near fracture tip. (a) HT-1 sample, and (b) HT-2 sample.

Fracture surfaces of crept samples are shown in Figure 9. The surfaces of grains could be observed on the fracture surfaces of HT-1 samples (Figure 9(a)); flat fracture surfaces and sharp cleavage edges could be identified (Figure 9(b)). By contrast, the fracture surfaces of HT-2 samples were dominated by rough surfaces and small dimple structures (Figure 9(c,d), indicating a mixture of intergranular and transgranular fracture. To further analyze the effect of SEGB on the crack propagation behavior during creep deformation, cross sections of fracture tips were examined and shown in Figure 10. At low magnification, cracks propagated mainly along the grain boundary in both samples. Cracks in HT-1 sample were straight and connected (Figure 10(b)), by contrast, creep damages along grain boundaries in HT-2 sample were often observed in segments (Figure 10(d)). Strain distributions are shown by EBSD KAM analysis (Figure 11); intergranular crack could be identified for HT-1 sample, and strains were mainly concentrated along grain boundary and at the triple junction (Figure 11(a)). By contrast, the strain distribution of HT-2 sample was more evenly dispersed (Figure 11(b)).

## 4. Discussion

Both HT-1 and HT-2 samples have almost identical tensile strength and ductility at 25°C (Table 1 and Figure 6); ductile transgranular fractures were dominant at room temperature (Figure 7(a,c)). However, when testing temperature was increased to 650°C, a significant hot ductility drop was observed for HT-1 sample. According to Figure 7(b), the facet cleavage surface and the limited amount of plastic deformation indicate that the failure mechanism was brittle intergranular fracture. The brittleness of HT-1 sample was even more pronounced under creep at 650°C as the creep fracture strain was only 0.03%. Cleavage edges on crept fracture surface (Figure 9(b)) and crack propagation along straight grain boundary (Figure 10(b)) indicate the vulnerability of straight grain boundary in HT-1 samples at elevated temperature. According to the local strain concentration at triple junctions of grain boundaries (Figure 11(a)), it is probable that HT-1 samples encountered an embrittlement at 650°C. The initiation of creep damage of HT-1 sample was a result of vacancy coalescence on grain boundaries perpendicular to the loading direction and severe strain concentration at grain boundaries. By contrast, the serrated grain boundaries in HT-2 sample could provide resistance against shear parallel to the grain boundary surface and creep damage were often observed in segments as shown in Figure 11(d).

This work has demonstrated that a simple post heat treatment could induce SEGB in a HEA fabricated by SLM. The serrations on grain boundary in HT-samples were attributed to the blocky irregular shaped γ′ precipitates formed during the slow cooling process, and they could pin the mobile grain boundaries as well as restricting grain growth, this phenomenon is known as the Zener drag effect [66–68]. The drag force from the coarse precipitates would hinder the migration of grain boundary, while those free from the precipitates could break free around the precipitates and resulted in serrated morphology (Figure 4(c)). The inhibition of grain growth from coarse precipitates explained why HT-2 sample possessed a smaller grain size than that of HT-1 sample. The difference in tensile yield strength between HT-1 and HT-2 at 25°C and 650°C could be associated with the size of γ′ precipitates. According to Jackson – Reed model [69], the strengthening contribution of γ′ precipitate could be inverse proportional to the average radius of the precipitate. With the slow cooling step in HT-2, the size of γ′ precipitates were coarser (Figure 5(e,f)) and would result in lower strengthening contribution from γ′ precipitate comparing with that of HT-1. On the other hand, improvement of tensile ductility in HT-2 sample agreed with those reported in previous studies [50,51], suggesting that serrated grain boundary could exhibit more steady plastic deformation and higher tolerance to intergranular cracking.

The enhancement of creep resistance by introducing serrated grain boundary in HT-2 samples was very noticeable. The beneficial effect of serrated grain boundary on creep properties can be rationalized by two aspects, the first is a decrease in minimum creep strain rate, and the second is the extension of the tertiary creep region. The minimum creep strain rate of HT-2 samples dramatically decreased by 96% compared with HT-1 sample (minimum creep strain rates of HT-1 and HT-2 samples were 6.4 × 10^−6^ s^−1^ and 2.4 × 10^−7^ s^−1^, respectively). Wu et al. [70,71] proposed a constitutive model that described creep behavior of alloys with serrated grain boundaries, and reported that minimum creep strain rate could be related to grain boundary morphology by a geometric factor ϕ, which was introduced to describe the interaction between local strains and grain boundary serrations, and it can be represented as:

(1) $$\begin{array}{c} \phi =\frac{2}{\sqrt{1+{\left(\frac{\pi h}{\lambda }\right)}^{2}}}-1 \end{array}$$

where λ = wavelength, and h= wave amplitudes of serrated grain boundaries. In this work, the geometric factors were also calculated to rationalize the contributions of grain boundary serrations. For HT-1 sample, ϕ equals 1 (λ»h for straight grain boundary). For HT-2 sample, the ϕ value is 0.017 (λ was 3.29 ± 0.38 μm and h was 1.69 ± 0.24 μm). Therefore, the value of ϕ of HT-2 sample could contribute to a decrease in creep rate by 98%, which is in good agreement with experimental analysis.

**Figure 12.**
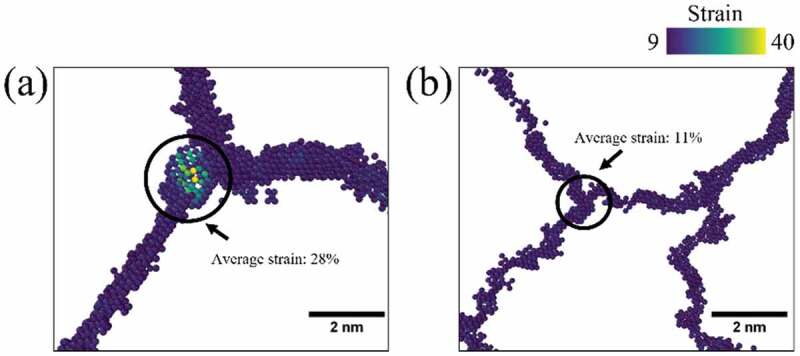
MD simulations of strain distribution on grain boundaries. (a) HT-1 sample, and (b) HT-2 sample. Severe localized strains on triple junction were clearly visible in HT-1 sample.

The extension of tertiary creep region was attributed to the retarded crack propagation in HT-2 sample due to grain boundary serration. Observations suggest that serrated grain boundary could serve as a crack deflector and retard connection of microcracks. Therefore, the resistance of intergranular cracking would be enhanced and resulted in a prolonged tertiary creep regime. As shown in Figure 11(b), the strain distribution of HT-2 sample during creep deformation was much more evenly dispersed with a reduced and localized strain concentration, previous study on serrated grain boundary suggested that undulation of grain boundary morphology could prevent localized strain concentration [72,73]. To further clarify the underlying mechanism of the effect of grain boundary morphology on strain accumulation during hot deformation, MD simulations have allowed the visualization grain boundary strain distribution during high temperature tensile loading (Figure 12). Simulation results demonstrated that severely localized strains at triple junctions, by contrast strains were more homogeneous distributed along serrated grain boundaries. Moreover, the average strains at the triple junctions (as indicated by circles) of HT-1 and HT-2 samples were calculated to be about 28% and 11%, respectively. This result indicates that SEGB could effectively alleviate strain concentration on grain boundary by more than 50%. This work has studied the mechanism of hot ductility drops of a HEA fabricated by SLM and demonstrated successfully that SEGB can be an effective approach to further improve high temperature mechanical properties.

## 5. Conclusions

The effect of serrated grain boundary on tensile and creep properties has been investigated for a high-entropy alloy (Al_8_Co_35_Cr_18_Ni_34_Ti_3_Nb_2_Zr_0.005_B_0.01_ (at.%)) fabricated by selective laser melting, the followings are findings of this work:

1. Serrated grain boundary could render a significant increase in tensile fracture strain and creep rupture life by a factor of 3.5 and 400, respectively.
2. The inferior ductility of straight grain boundary at 650°C was attributed to the grain boundary embrittlement caused by strain concentration at triple junctions and fast intergranular crack propagation.
3. The slow cooling process of the post heat treatment could coarsen the γ′ particles and encourage interactions with mobile grain boundaries to from serrated grain boundaries.
4. Analysis has indicated that serrated grain boundary could distribute strains more evenly than that of straight morphology. Serrated grain boundaries could reduce local strain concentration and provide resistance against intergranular cracking.
